# Exploring competing experiences and expectations of the revitalized community health worker programme in Mozambique: an equity analysis

**DOI:** 10.1186/s12960-015-0044-0

**Published:** 2015-09-01

**Authors:** Celso Soares Give, Mohsin Sidat, Hermen Ormel, Sozinho Ndima, Rosalind McCollum, Miriam Taegtmeyer

**Affiliations:** Department of Community Health, Faculty of Medicine, University Eduardo Mondlane, Av. Salvador Allende no.702, Maputo, Mozambique; Department of Health, Royal Tropical Institute, Amsterdam, Netherlands; Department of International Public Health, Liverpool School of Tropical Medicine, Liverpool, UK

**Keywords:** Community health workers, Equity, Community, Mozambique

## Abstract

**Introduction:**

Mozambique launched its revitalized community health programme in 2010 in response to inequitable coverage and quality of health services. The programme is focused on health promotion and disease prevention, with 20 % of community health workers’ (known in Mozambique as Agentes Polivalentes Elementares (APEs)) time spent on curative services and 80 % on activities promoting health and preventing illness. We set out to conduct a health system and equity analysis, exploring experiences and expectations of APEs, community members and healthcare workers supervising APEs.

**Methods:**

This exploratory qualitative study captured the perspectives of a range of participants including women caring for children under 5 years (service clients), community leaders, service providers (APEs) and their supervisors. Participants in the Moamba and Manhiça districts, located in Maputo Province (Mozambique), were selected purposively. In total, 29 in-depth interviews and 9 focus group discussions were conducted in the local language and/or Portuguese. A framework approach was used for analysis, assisted by NVivo10 software.

**Results:**

Our analysis revealed that health equity is viewed as linked to the quality and coverage of the APE programme. Demand and supply factors interplay to shape health equity. The availability of responsive and appropriate services led to tensions between community expectations for curative services (and APEs’ willingness to perform them) and official policy focusing APE efforts mainly on preventive services and health promotion. The demand for more curative services by community members is a result of having limited access to healthcare services other than those offered by APEs.

**Conclusion:**

This study highlights the need to pay attention to the determinants of demand and supply of community interventions in health, to understand the opportunities and challenges of the difficult interface role played by APEs and to create communication among stakeholders in order to build a stronger, more effective and equitable community programme.

## Introduction

Since independence in 1975, Mozambique has promoted a health policy based on the principles of broad and equitable access to health services through sustained expansion of the primary healthcare system. This included the introduction of the community health worker programme in 1978 as a strategic solution to existing constraints of limited access to healthcare services by the rural population [1]. Community health workers are known in Mozambique as Agentes Polivalentes Elementares (APEs), meaning “essential [or elementary] multi-purpose agents”, thus highlighting the intended focus on providing primary healthcare services to remote rural communities [2, 3]. However, the 16-year civil war (1976–1992) damaged the health system, negatively impacting not only facility-based healthcare services but also the APE programme as it impeded appropriate supervision of, and technical support to, APEs [4, 5].

Community health programmes as a means to accelerating progress towards the Millennium Development Goals (MDGs) have been embraced in many developing countries [6–9], including Mozambique. The revitalized APE national programme was rolled out in Mozambique in 2010 as a means of increasing the coverage (estimated to be below 50 %) and quality of primary healthcare [10]. It is focused on health promotion and disease prevention, with official guidelines indicating 80 % of APE’s time should be spent on these activities and only 20 % on curative services [11]. The 4-month APE training reflects this package of preventive, promotive and curative services. The training on curative care is limited to testing and treating malaria, diagnosing and treating diarrhoea (oral rehydration only) and providing antibiotic treatment for acute respiratory infections in children, providing first aid and being able to detect health danger signs in children, adults and pregnant women [12]. The policy states that APEs should be placed to serve 500 to 2000 inhabitants (depending on population density and geographical coverage), and APEs should ideally be working between 8 km and 25 km from the health facility of their reference – far enough to cater to underserved populations and close enough to allow appropriate supervision and support from the health system staff [13]. APEs are volunteers who sign an agreement, describing their right to an allowance and access to free healthcare at the local health centre. Although the allowance is not based on performance, in practice, it may be withheld if APE reports are incomplete or delayed.

Community health programmes have also been seen as a potential way of improving equity of healthcare. In order to ensure equity, any health service must be accessible, acceptable and of equal quality for all [14], regardless of a person’s bio-social determinants, such as place of residence, race, occupation, gender, religion, level of education, socioeconomic position, social capital, age, sexual orientation or presence of disability [15]. In Mozambique, the extent of equity of service coverage and quality is uncertain, and the impact of the APE programme on equity is unclear. A wide range of contextual factors influence the equity of the programme including material circumstances, psychosocial and behavioural factors, biological (such as genetics, age and sex) and health system factors [16]. These factors can be characterized as demand-side or supply-side determinants [17, 18]. On the one hand, demand-side determinants influence health-seeking behaviour and access to health services (for example, if sociocultural beliefs limit health-seeking behaviour, if the APE is hard to reach or if a service is not acceptable to the community). On the other hand, supply-side determinants are aspects related to the health system and its policies and implementation arrangements (for example, APEs being located within a certain distance of a facility (8–25 km) or being trained to deal primarily with childhood illnesses). These factors are likely to affect poor and other vulnerable groups more than others, as costs of access, lack of information and cultural barriers impede them from benefiting from public health efforts [19]. Finding the balance between demand and supply of health services within budgetary constraints continues to be a big challenge in Mozambique. APEs are uniquely placed to comprehend communities, spread health promotion messages and help link them to services. However, their very proximity and “embeddedness” within communities places them in a difficult negotiating space. On the one side, they are faced with an inability to satisfy demands from the community for more curative care. On the other, they need to negotiate with the health system to allow them to provide more curative services, while not neglecting health prevention and promotion priorities. This paper aims to explore the tensions and opportunities provided by the interface role of these “close-to-community” (CTC) providers through the lens of an equity analysis considering equal access to services for equal need, equal utilization of services for equal need and equal quality of care for all, based upon Whitehead’s definition of equity [20].

## Methodology

Qualitative methods were used for this exploratory study in order to capture the perceptions and perspectives of a range of participants including community members, community leaders, service providers (APEs) and their supervisors [21]. Two districts in Maputo Province, Manhiça and Moamba, were selected in collaboration with the Ministry of Health and an advisory group of key stakeholders in community health. Both districts are mainly rural and have an established revitalized APE programme. Their health networks, including both facility-based and community-based health services, remain insufficient to meet the needs of the population, and the epidemiological situation is dominated by malaria, acute respiratory infection, diarrhoeal diseases, sexually transmitted infections and HIV infection [22]. Community entry was achieved through the Maputo Provincial Health Directorate, who supplied a letter for the district APEs’ coordinator. The research team planned their activities together with these coordinators to ensure these were realistic. The health facilities of reference for the APEs were used as a starting point.

Two researchers worked together and conducted a total of 29 in-depth interviews (IDIs) with APEs, community leaders and APE managers and 9 focus group discussions (FGDs) with mothers of children under 5 who had used APE services. They held daily debriefing meetings to discuss data collected and to share initial impressions as well as to triangulate and clarify diverse or contradictory findings. Participants were selected purposively to ensure representation based on geographical location, distance to health facilities, gender, age and job experience. Both district supervisors were selected for inclusion and they, in turn, identified health facility supervisors. APEs, both those reporting to the selected supervisors and those who were not, were sampled based on willingness to take part and availability at the time of interviewing. Community leaders and mothers were convenience-sampled with support from APEs and resided within a limited radius of the APE’s work station. Supervisor interviews were held in Portuguese, recorded and transcribed verbatim. The remaining interviews were largely conducted and recorded in the local languages of Ronga and Xi-Changana, requiring translation to Portuguese as they were transcribed. Since the local language is spoken and not written, tools were in Portuguese and their use in the local language required thinking through and consensus by the team. Translations and transcriptions were done by the research team with considerable knowledge of the local language, Portuguese and English. Additionally, during this process, all team members worked together to discuss, clarify and confirm their understanding of the data. In-depth interviews were used with managers, community leaders and APEs to elicit sufficient depth of understanding about the APE programme and the feelings and perceptions of the individuals, allowing sensitive areas to be probed. Meanwhile, FGDs were undertaken with mothers of children under 5 years to use group interaction to generate findings to help understand community norms, common health issues and the need for, access to and use of healthcare services [23–25].

A framework approach was employed for analysis, with emerging themes used to develop a coding framework, based on team consensus [26]. Codes on equity included perceptions related to the quality of the service, the access to and coverage of APE services and the availability of referral points. We also included codes on APEs feeling responsible and responsive on community engagement and governance and on formal links to the health system and its supervisors. Analysis was supported by the use of the qualitative analysis software Nvivo (v10) for coding of transcripts. These were then further analysed by running queries according to the main codes and sub-codes, while more complex queries looked at sub-groups. Query results were summarized in narratives for each theme and sub-theme, which in turn were reviewed, discussed and adjusted. A full report of the larger context analysis of which this forms a part with extensive quotes and draft narratives was produced and published [27].

Ethical clearance was obtained from the ethics committee at the Royal Tropical Institute (KIT) in the Netherlands and the Institutional Bioethics Joint-Committee of Maputo Central Hospital and Faculty of Medicine of University Eduardo Mondlane (reference number CIBSFM&HCM07/2013). Administrative approval was obtained from the Maputo Provincial Health Directorate and the District Health Directorates of Manhiça and Moamba. The study implementation adhered to good research practice, including obtaining informed consent of study participants and maintenance of anonymity and confidentiality of all data. To ensure the anonymity of respondent quotations, district and health facility supervisors were grouped into a single category called “APE managers”.

## Results

We interviewed 18 APEs, 5 APE managers and 6 community leaders, while 67 primary care givers (mostly mothers) of children under 5 years old participated in one of nine FGDs (see Table 1). The “Results” section presents three key themes that emerged from the analysis, and they demonstrate the interplay between community perspectives (demand side) and policy and practice (supply side). Each has implications for the evolving role of APEs as they build bridges between rural communities and health systems. In the first theme, health equity is seen as linked to the quality and coverage of service. In the second theme, the availability of responsive and appropriate services is covered, highlighting the tensions between community demands for curative services and the official APE policy focus on promotive and preventive services. Finally, the third theme explores accountability and ownership, that is, whom the APEs are responsible for and responsive to.

**Table 1:** Sociodemographic profiles of study participants (in absolute numbers)

		APEs	APE managers	Community leaders	Mothers
Data collection method		IDI	IDI	IDI	FGD
Total sample		*n* = 18	*n* = 5	*n* = 6	*n* = 9
Districts	Moamba	10	3	2	20
	Manhiça	8	2	4	21
Gender	Male	10	4	5	41
	Female	8	1	1	0
Age (years)	18–25	9	1	0	11
	26–35	3	3	0	17
	35–44	5	0	0	4
	>45	1	1	6	8
Marital status	Married	6	5	6	32
	Single	11	0	0	7
	Divorced	1	0	0	2
Education	None	0	0	2	12
	Primary	10	1	3	25
	Secondary	8	4	1	3

### Quality, access and coverage

APEs in both districts were highly valued within their communities for the availability and quality of services they provided and described themselves as striving to provide services to all people based on need and without discrimination. Their managers, who are facility-based staff, thought of them as a “third arm” of the health system. However, all respondent types recognized the difficulties faced by APEs in the context of resource constraints.

#### Perceptions of the quality of community health services

In both districts, community respondents agreed that APEs served as a link between communities and health facilities and that the quality of services provided by APEs was good and was helping communities reduce disease by prevention and curative activities. They appreciated APEs’ persistence in promoting community health through basic knowledge and good hygiene practices:

“(…) Another thing that makes me considering it a good program is because the APEs teach us many things in our homes. They teach us how to take care of our homes, water and food in order to not catch diseases.” (Mother, 34 years old, female, Moamba).

Community members felt that APEs provide effective treatment, responsive to the request for care from community members:

“What makes us say their services are of a good quality is that you never see someone going to an APE and not being attended. If you go while you are ill you will get medication and come back better.” (Community Leader, 57 year old, male, Moamba).

These perceptions about the quality of work carried out by APEs generated confidence and satisfaction among community members. The main community complaints regarding the quality of APEs’ work relate to the low numbers of APEs working in each community and stock-outs of medicines. The first issue was mirrored by APEs as well, who acknowledged long working days with considerable distances to cover, rather than suggesting their workload was too high. Stock-outs of drugs were reported to occur frequently and may be related to the supply system logistics, with new supplies based on a monthly supply kit and stock use reports for malaria tests. The quality of care provided by APEs was acknowledged by most supervisors as well, who argued that despite some constraints, APEs in general provided a good service:

“Well, the quality of service is good, [he] may miss one or another aspect because the procedures and health programs are not static, they are dynamic. [APEs] are not in time to keep up … but the work is of quality.” (APE Manager 1).

APEs themselves had positive perceptions regarding the quality of care they provided, as they had not received complaints from the community and received requests to provide more services. People had improved following their treatment, which was recognized by one APE as an indicator of quality:

“I think it’s good because there are people who appear here as serious[ly ill], I give medicine and [they] improve. Others I give a referral guide (…) but do not go (…) because they have already improved. When I respond to someone’s request and then the person improves, to me that means quality work.” (APE, 23 years old, female, Moamba).

#### Access and coverage

Access to healthcare services was addressed to explore the ease or difficulty with which community members have access to the scope of these services and the ease or difficulty for APEs in reaching communities. This can manifest itself in the form of discriminatory practices but also in terms of geographical distances. Regarding equity in access, all participants agreed there was no differentiation or discrimination between members of the community, as this APE explained:

“When I was training I swore I would treat all people in my community without choosing anyone, also because if I do not treat people here in my community it is the same as abandoning my own family…” (APE, 22 years old, female, Manhiça).

Many community members stated that they found APEs more approachable than health facility staff, easier to talk to, less judgemental and more responsive to their needs.

One of the problems frequently described as influencing access to health services was that of distance, inaccessibility and lack of transportation. Participants referred both to distances within the community, regarding the extent and dispersion of the homes of community members, and to the prohibitive distance to health facilities. Limited transport options and difficulties in accessing households were felt by almost all participants to exert a great influence over the extent to which APEs can visit homes within their catchment areas. Several APEs and mothers of children under 5 mentioned this as a major difficulty:

“Here we have the locality 1, 2 and 3, but I hardly ever went to the interior of the neighbourhood 3 because the distance is too far and the access conditions are more difficult, especially when riding a bicycle.” (APE, 42 years old, female, Manhiça).

The issue of distances was also reported as challenging when collecting new supplies, because APEs must walk long distances while carrying a heavy load on their head:

“What hurts me most here is the distance, for example, when the kit arrives at the health unit [facility name], it is a kit that weighs about 20 kg or 18 kg, so I have to walk out of here to go to [facility name]and take the box and walk that whole distance of 20 km with the box on my head to and from, it is not easy…and then even worse when I have to go from house-to-house on the same day or next day.” (APE, 36 years old, male, Manhiça).

### The availability of responsive and appropriate services

The community demand for curative and other services not formally seen as part of APEs’ tasks was a common discourse and is in part driven by low coverage and difficulties in gaining access to the health facilities. Mothers and community leaders in both districts emphasized the need for growth monitoring, immunization, maternity care, antenatal care services and an increased range of other (particularly paediatric) curative services provided by APEs. This was due to the current challenges involved with using these services at the health facility due to lack of transportation and money to pay for it. Mothers felt that APEs should offer these services to minimize the cost for clients and reduce distances:

“We also have growth monitoring problems; sometimes the health professionals say that they will come but they don’t appear, so if we had growth monitoring here there would not be a problem. And it is difficult to carry a child to health facilities especially if the child already is more than a year old, so this is what makes hard work to us.” (Mother, 29 years old, female, Manhiça).

From the perspective of the community, it was seen as a practical solution to request that the APE receives training to provide a wider range of curative services. The standard APE package was regarded as insufficient by community members, by APEs and by some APE managers, who argued that it cannot address all health issues in the community, as pointed out by one manager:

“I think it could be very good if the Ministry of Health invested in training APEs in tasks demanded by the community, (now) the APE cannot address some diseases because he doesn’t have training for it”. (APE Manager 2).

The same view was stressed by APEs, who feel caught between demand and supply:

“Sometimes is difficult to me when a community come to me to have a health service and I tell them that this disease I cannot treat. I would like to have more training to avoid this and help much more my community”. (APE, 36 years old, male, Moamba).

Regarding the types of care provided by APEs, participants made reference to the treatment of diseases such as diarrhoea, malaria and respiratory infections, while clients with other diseases are referred:

“Many children who come here I have to treat for problems related to diarrhoea, malaria, fever and breathing problems. Sometimes people appear with ‘rheumatism’ problems, especially older people always complain of rheumatism”. (APE, 22 years old, female, Manhiça).

“…I went there yesterday because I did not feel good, my body was aching and I felt headaches. When I arrived he said that was not a problem he could solve…so he gave me paracetamol and told to go to the health facility.” (Community Leader, 58 years old, male, Moamba).

“Mothers go to Checua only because of the growth monitoring. But our APE says that she doesn’t know and she doesn’t have the equipment for that, so if they could increase her knowledge it would help.” (Community Leader, 78 years old, male, Manhiça).

This issue emerged as central in the Manhiça district. It was clear that communities perceive the APEs to be a type of “doctor” and that their health post (which is officially supposed to have been shut down as part of the revitalized programme) represents an extension of health facilities to communities, leading to demand for increased capacities of their “doctor”:

“Well, I think what they would need to learn more is about how to apply injections and take blood tests to see what disease the person has. There is no medicine for paralysis, for hypertension, so why not teach them? That is what we think should increase.” (Community Leader, 54 years old, male, Moamba).

“Yes we would like you to have drugs for asthma and… for all kinds of diseases we have.” (Mother, 33 years old, female, Manhiça).

### Accountability and ownership of APEs

The APE programme was widely regarded as a bridge between communities and the health system, and this was described as a formal expectation of the role by APEs and managers but less so by community members:

“The people from the health facilities told us that we must serve the community and be a link between the community and the health system.” (APE, 33 years old, female, Moamba).

“Since the time of recruitment and during the training we stress that the aim of the APE program is to work in the community… as well as to serve as a link between the community and health facilities.” (APE Manager 3).

Data from APE interviews as well as interviews with mothers, community leaders and managers reveal that this interface role created a sense of dual responsibility. APEs feel responsible to the health system on the one hand, with its reporting demands in exchange for training and supplies for APEs, and to the community on the other hand, as they were selected and are supported by the community and hence feel an intense loyalty towards them.

Both community members and the APEs themselves view APEs as “community doctors” with communities taking a direct supervisory role of their “doctors” as described by one community leader:

“The APE is very committed with our health problems and what I most like from him is that he accepts the criticism, when you tell him that this is not right he asks you in which way should he proceed.” (Community Leader, 49 years old, female, Moamba).

APEs described feeling caught between expectations and boundaries, as they were selected to serve the communities at all times and to be easily accessible and responsive:

“*My work must never end for the community*. I’ve got to meet the people at the time they arrive and need my help.” (APE, 43 years old, male, Manhiça).

Responsiveness to community expectations and supervision thus serve as key factors in the legitimacy and social acceptance of APEs, contrasting with the relative remoteness of guidance and supervision from the health facility and supervisory staff. APEs described agency in the way they construct their roles to satisfy both sides. Some described spending far longer periods of time than formally foreseen on curative tasks when treating individuals, while passing health promotion messages to large groups of people enabled them to meet their 20 % curative and 80 % promotive target. Such co-construction of reporting mechanisms which suit the community demands and health system requirements appeared to satisfy all parties and demonstrates strategic agency by APEs in balancing the expectations of both communities and health systems.

## Discussion

Our findings illustrate that APEs are appreciated by communities, who regard them as “community doctors” providing bridges to the health system. However, in the context of resource constraints and a weak health system, they face tensions in their ability to be fully responsive to the needs and priorities of adults and children from remote rural areas with limited or no access to healthcare. Additionally, APEs find themselves caught between community demands for broader curative services while the official policy largely limits their focus to activities related to promotion of health and prevention of diseases. APEs are responsive and accountable to both community and health system officials (supervisors) which between them have contrasting demands and in some cases show agency in co-constructing a middle path acceptable to all.

As the APE programme extends the reach of healthcare, it is expected that bringing services closer to the homes of their clients will be more likely to reach the disadvantaged [28]. This expectation was fulfilled to some extent in our study, with improved equity of health services described through increased access to health services provided by APEs compared with services provided at the health facility level, greater acceptability of APE services compared with facility services and community perceptions of quality APE service provision. However, despite these improvements, limitations in terms of equity remain when demand- and supply-side dimensions were explored further. Delays in seeking healthcare remain common with both demand- and supply-side factors implicated and more so among poorer rural populations [29]. Leading challenges described are responsiveness of APEs to the community’s demands and conflict in accountability for APEs between community and the formal health system. Given the tension between providing a greater range and quantity of curative services at the community level and the formal health system’s emphasis on preventive care within training and reporting, there is the possibility that the quality of services may be undermined if the APEs were to take on multiple additional roles and curative tasks in response to community demands that they are ill-prepared for and under-supported to carry out. The weak capacity to effectively follow up, monitor and supervise APEs activities; existing norms that allow the prescription of some drugs by APEs; and limitations of training of APEs in curative activities [30] will further affect quality and could potentially lead to inappropriate use of antimicrobial drugs and drug resistance. The current selection criteria for APEs include a relatively low educational level and an evaluation carried out following the first training of APEs by the Ministry of Health (MoH) and partners suggest that 31.8 % of APEs experienced difficulty in understanding the training content due to the unfamiliarity with biomedical terminology and volume of lessons [30]. Despite the APE programme and findings presented from this study, national health service coverage remains limited in Mozambique [10]. This results in persistent pressure from communities for the MoH to instruct and deploy APEs in areas outside established limits. Communities therefore continue to regard APEs as a solution to their health concerns and curative needs, and the optimal balance between curative and promotive tasks remains a matter of perspective that will require further deliberations among stakeholders, including community members.

Linking communities and health systems is an explicit role of the APEs in Mozambique and CTC providers elsewhere [31, 32]. To make this work well, it needs to be conceptualized through a system-thinking lens and guided by lessons learned from task-shifting and decentralized HIV care [33]. Specific attention needs to be paid to how the design of the programme can affect community health worker (CHW) performance [34]. The voices of APEs are key in this process and underscore the importance of continued communication between the health system and communities [35, 36]. APEs’ bridging role, connecting communities to the health system, provides a number of opportunities as well as challenges. APEs are clearly central to health systems and should be recognized as such. They are uniquely placed to understand the cultural and attitudinal aspects of healthy practices and health-seeking behaviour and to act as cultural brokers in this and in generating demand for services [30, 37]. They also face significant challenges through their proximity to the communities and the difficulties of their interface role [8, 34, 38].

Our study has several limitations. APEs were involved in the selection and recruitment of participants for FGDs with mothers and the IDIs with community leaders; this may have led to bias in these respondents’ answers. APEs tended to select participants living in close proximity to where the discussions were held (the village with the health post to which the APE was attached) and, therefore, also lived closer to the health post and had easier access to the APEs and their services. Rural areas in Mozambique, even in our two districts, are hard to access by bicycles, walking and 4 × 4 vehicles, and this meant that respondents were located no more than a 5- to 6-h drive from the district headquarters. Additionally, respondents selected by APEs may have been those they knew well or with whom they had a good relationship. To overcome this limitation, we focused our questions on the overall programme and not on individual APEs. Additionally, we triangulated information on client experiences across the two districts and included providers’ views. The decision to limit interviews with service users to women with children under 5 was made in view of the focus of the APEs’ programme on children’s health, and this presents a limitation given that many APEs provide curative services to adults as well. The level of education of community members was relatively low and influenced their level of understanding of issues addressed during data collection as well as the need for additional layers of translation, from local languages to Portuguese, with a potential loss of fidelity in transcribing. Both of the data collectors were male. The supervisors and community leaders tended to be male, although overall our respondents were predominantly female and this is likely to have had an impact on their responses and perceptions. Although we did not set out to explore this deliberately, we found very few gender differences in the results, with female/male respondents (APEs and others) having similar views and perspectives. A fuller gender analysis and exploration of the impact of gender on community and APEs’ perceptions of the interface role is required.

## Conclusions

We conducted a qualitative study in two districts in a southern province in Mozambique in order to explore the interface role of APEs between communities and health systems from multiple perspectives. The findings describe equity improvements in terms of access, acceptability and community perceptions of quality of health service provision. However, findings suggest a disconnect between the needs of the populations and how the APE policy was originally conceptualized. This situation is generated by the differing views of community and policy makers on what is important in terms of curative services. The view that the APE is like a “community doctor”, a lack of health facilities and a lack of transport means that community members often turn to APEs for treatment and APEs try their best to be responsive to this, while still trying to ensure (or constructing a narrative that implies that) they also meet health system expectations to devote 80 % of their time to promotive activities. This study highlights the need to pay attention to the determinants of demand and supply of community interventions in health and to create strong communication among stakeholders to prevent undermining the APE programme.
